# Diagnostic Utility of Intratumoral Susceptibility Signals in Adult Diffuse Gliomas: Tumor Grade Prediction and Correlation with Molecular Markers Within the WHO CNS5 (2021) Classification

**DOI:** 10.3390/jcm14114004

**Published:** 2025-06-05

**Authors:** José Ignacio Tudela Martínez, Victoria Vázquez Sáez, Guillermo Carbonell, Héctor Rodrigo Lara, Florentina Guzmán-Aroca, Juan de Dios Berna Mestre

**Affiliations:** 1 Department of Dermatology, Dentistry, Radiology and Physical Medicine, Faculty of Medicine, University of Murcia, Av. Buenavista, 32, 30120 El Palmar, Spain; vivaz1820@gmail.com (V.V.S.); florentina.guzman@gmail.com (F.G.-A.); juandeberna@um.es (J.d.D.B.M.); 2 Radiology Department, Virgen de la Arrixaca University Hospital, Ctra. Madrid-Cartagena, s/n, 30120 El Palmar, Spain; guilleclc@gmail.com; 3 Pathology Department, Virgen de la Arrixaca University Hospital, Ctra. Madrid-Cartagena, s/n, 30120 El Palmar, Spain; rodrigohector@outlook.es

**Keywords:** glioma, susceptibility weighted imaging, IDH, ITSS, 1p/19q, CDKN2A/B

## Abstract

**Background/Objectives**: This study evaluates intratumoral susceptibility signals (ITSS) as imaging markers for glioma grade prediction and their association with molecular and histopathologic features, in the context of the fifth edition of the World Health Organization Classification of Tumors of the Central Nervous System (WHO CNS5). **Methods**: We retrospectively analyzed patients with adult diffuse gliomas who underwent pretreatment magnetic resonance imaging. ITSS were semiquantitatively graded by two radiologists: grade 0 (no signal), grade 1 (1–5), grade 2 (6–10), and grade 3 (≥11). Relative cerebral blood volume (rCBV) and tumor volume were also obtained. Histopathologic features included tumor grade, Ki-67, mitotic count, necrosis, microvascular proliferation, and molecular alterations (isocitrate dehydrogenase [IDH], 1p/19q, cyclin-dependent kinase inhibitors 2A and 2B [CDKN2A/B], and p53). Regression models predicted tumor grade (low: 1–2, high: 3–4) using ITSS, tumor volume, and rCBV. ROC curves and diagnostic performance metrics were analyzed. **Results**: 99 patients were included. ITSS grading correlated with rCBV, tumor volume, mitotic count, Ki-67, and tumor grade (*p* < 0.001). ITSS grades 0–1 were associated with oligodendrogliomas and astrocytomas (*p* < 0.001), IDH mutations (*p* < 0.001), and 1p/19q co-deletions (*p* = 0.01). ITSS grades 2–3 were linked to glioblastomas (*p* < 0.001), necrosis (*p* < 0.001), microvascular proliferation (*p* < 0.001), and CDKN2A/B homozygous deletions (*p* = 0.02). Models combining ITSS with rCBV and volume showed AUC of 0.94 and 0.96 (*p* < 0.001), outperforming univariate models. **Conclusions**: Semiquantitative ITSS grading correlates with key histopathologic and molecular glioma features. Combined with perfusion and volumetric parameters, ITSS enhance non-invasive glioma grading, in alignment with WHO CNS5.

## 1. Introduction

Diffuse gliomas are the most common primary malignant central nervous system (CNS) tumors in adults; isocitrate dehydrogenase (IDH)-wildtype glioblastoma is the most frequent and aggressive subtype [1]. The fifth edition of the World Health Organization (WHO) Classification of Tumors of the CNS (WHO CNS5) redefined adult diffuse gliomas by emphasizing the diagnostic and prognostic significance of molecular markers, including IDH mutations, 1p/19q co-deletion, and deletions of cyclin-dependent kinase inhibitors 2A and 2B (CDKN2A/B), which are commonly used in clinical practice [2,3].

Given the prognostic significance of these genetic alterations, there is an increasing need for imaging biomarkers that can non-invasively predict tumor molecular profiles and aggressiveness. In this context, the semiquantitative assessment of intratumoral susceptibility signals (ITSS) has emerged as a promising imaging biomarker [4,5,6,7,8]. ITSS appear on susceptibility-weighted imaging (SWI) as low-signal-intensity dot-like or linear foci within tumors and may offer valuable insights into tumor biology, grading, and prognosis by providing a non-invasive approach to assessing tumor characteristics [9,10,11,12,13,14].

Prior to the introduction of the WHO CNS5 classification in 2021, ITSS were successfully applied for glioma grade prediction, highlighting their complementary value when interpreted alongside classical radiological markers of aggressiveness [4,5,6,10]. In addition, ITSS were also combined with perfusion and diffusion imaging to predict IDH and 1p/19q status in lower-grade gliomas [10,15]. Since 2021, studies have integrated ITSS with relative cerebral blood volume (rCBV) to predict tumor grade in IDH-mutant astrocytomas, demonstrating significant outcomes with satisfactory results [5,12]. However, these investigations have often focused on limited glioma subtypes and have not integrated a broader spectrum of molecular markers, such as CDKN2A/B deletions, which are now essential to the current diagnostic framework.

In this study, we hypothesized that semiquantitative ITSS grading is associated with histopathological and molecular features in adult diffuse gliomas and, therefore, can be used to non-invasively predict tumor grade, as defined by the WHO CNS5 classification. Our aim was to evaluate the diagnostic performance of ITSS for tumor grade prediction, as well as to assess the correlation of ITSS with key radiological, histopathological, and molecular markers, including rCBV, tumor volume, mitotic activity, Ki-67 index, necrosis, microvascular proliferation, IDH mutation, 1p/19q co-deletion, CDKN2A/B deletion, and p53 expression.

## 2. Materials and Methods

### 2.1. Patients

This single-center retrospective study was approved by the ethics and research committee of Virgen de la Arrixaca University Hospital, Murcia, Spain. To minimize selection bias, all patients with a pathologically confirmed diagnosis of adult diffuse glioma between January 2022 and April 2024 were included. The exclusion criteria were: (1) age under 18 years, (2) unavailable or incomplete histological diagnosis or molecular testing results, and (3) incomplete or technically inadequate magnetic resonance imaging (MRI).

### 2.2. Imaging Acquisition

An MRI was performed before biopsy or resection on a 1.5 T system (Signa Explorer; General Electric (GE) Healthcare) using our institutional glioma-specific MR imaging protocol. The sequences analyzed in this study were the following: 3D T2 susceptibility-weighted angiography (3D SWAN), dynamic susceptibility contrast (DSC) perfusion imaging, and 3D T1 fast-spoiled gradient-recalled-echo brain volume (3D T1 BRAVO). The 3D SWAN sequences were acquired without contrast administration (echo time [TE] = 50 ms, repetition time [TR] = 75.1 ms, matrix = 228 × 224, field of view [FOV] = 240 mm, section thickness = 3 mm, flip angle = 25°). For DSC perfusion imaging (gradient echo echo-planar imaging, TE = 40 ms, TR = 1900 ms, matrix = 128 × 26, FOV = 240 mm, section thickness = 5 mm, number of slices = 22, flip angle = 60°), we used 1 mmol/mL gadubotrol (Gadovist, Bayer Healthcare) as a contrast agent. The dose was weight-dependent (7.5 mL/kg) and was injected as an intravenous bolus at a flow rate of 5 mL/sec, followed by a saline flush of 20 mL (0.9% NaCl). Post-contrast 3D T1 BRAVO sequences (TE = 3 ms, TR = 7.7 ms, inversion time [TI] = 450 ms, matrix = 256 × 256, FOV = 256 mm, section thickness = 2 mm, flip angle = 12°) were acquired after DSC perfusion imaging.

### 2.3. Histopathological and Molecular Study

Histopathological evaluation was conducted by expert neuropathologists in accordance with the WHO CNS5 classification and grading system. All samples were obtained through surgical resection or biopsy. Hematoxylin-eosin staining was used to assess mitotic count, presence of necrosis, and microvascular proliferation. Immunohistochemical techniques were performed to determine the Ki-67 index (clone MIB-1; Dako Agilent, Santa Clara, CA, USA) and p53 mutation (clone DO-7; Dako Agilent, Santa Clara, California, USA). Analyses were conducted on the Dako Omnis platform (Dako Agilent, Santa Clara, CA, USA). IDH status, 1p/19q co-deletion, and CDKN2A/B deletions were performed using multiple-ligation-dependent probe amplification (P088-C2 kit, MRC-Holland, Amsterdam, The Netherlands). Fragment analysis was conducted with an ABI 3500 Genetic Analyzer (Applied Biosystems, MA, USA), and the results were normalized using Coffalyser software v240129.1959 (MRC-Holland, Amsterdam, The Netherlands).

### 2.4. Data Collection

**Figure 1:**
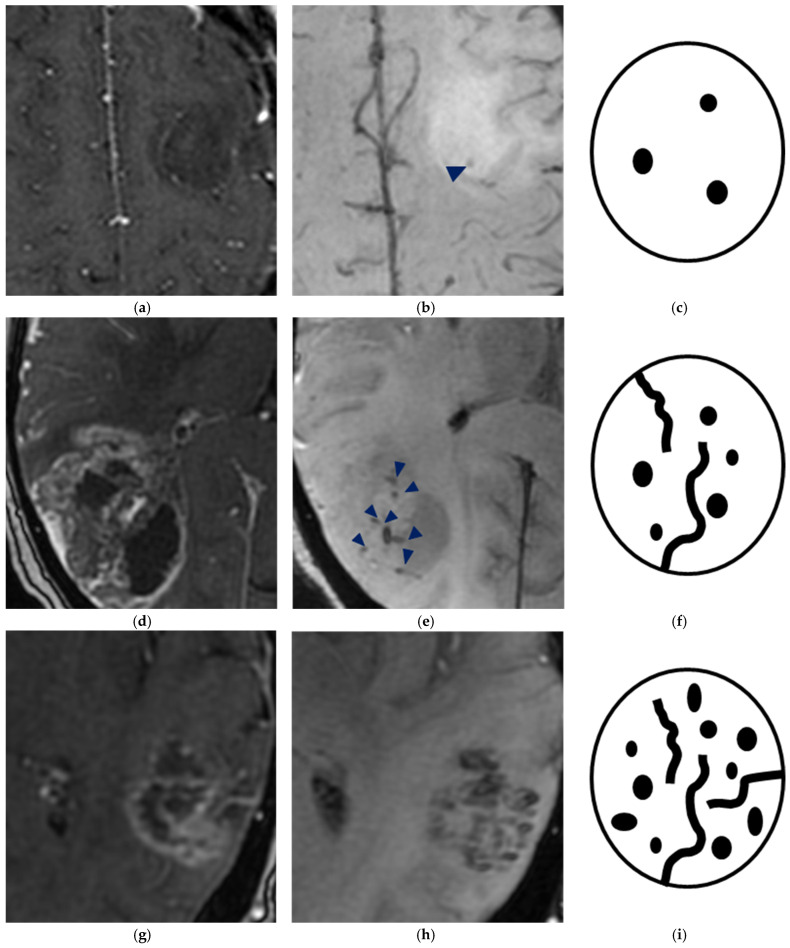
Semiquantitative ITSS assessment: (**a**–**c**) grade 1: 1–5 dot-like or linear ITSS (arrowhead); (**d**–**f**) grade 2: 6–10 dot-like or linear ITSS (arrowheads); and (**g**–**i**) grade 3: ≥11 dot-like or linear ITSS. MRI sequences: postcontrast 3D T1 BRAVO (**a**,**d**,**g**); 3D SWAN (**b**,**e**,**h**).

All data were collected by the study’s coordinating radiologist (J.I.T.M.). MRI studies were independently evaluated by two neuroradiologists, with 30 (V.V.S.) and 10 (G.C.) years of practice experience who were blinded to both pathological and molecular diagnoses, having only been informed that the tumor was categorized as a diffuse glioma. Using 3D SWAN sequences, ITSS were classified into four grades, as previously described [16]: 0 (no ITSS), 1 (1–5 dot-like or linear ITSS), 2 (6–10 dot-like or linear ITSS), and 3 (≥11 dot-like or linear ITSS) (Figure 1). Posteriorly, interobserver variability was analyzed, and discrepancies were resolved through consensus. Tumor volume was obtained from the contrast-enhancing parts of the 3D T1 BRAVO images using the ABC/2 formula [17,18]. DSC perfusion maps were used to obtain rCBV values, which were normalized to contralateral normal-appearing white matter by placing a region of interest (ROI) of similar size in the corresponding area of the opposite hemisphere. Leakage correction was applied. Measurements were performed using the GE AW Server (v3.2, Ext. 4.8).

All histological diagnoses and molecular profiles were reviewed by an expert neuropathologist (H.R.L.) with 10 years of experience in brain tumor pathology. Based on the WHO CNS5 classification, patients were categorized into low-grade glioma (LGG, CNS WHO grades 1–2) and high-grade glioma (HGG, CNS WHO grades 3–4) groups. Necrosis, microvascular proliferation, IDH mutation status, CDKN2A/B deletion, 1p/19q co-deletion, and p53 alterations were treated as binary variables (present/absent). Positive IDH mutations were further categorized based on the specific mutation type, while positive CDKN2A/B alterations were classified by deletion type (homozygous or heterozygous).

### 2.5. Statistical Analysis

Spearman’s correlation was used to study the relationship between the ITSS grade and quantitative variables, including tumor volume, rCBV, CNS WHO grade, Ki-67 index, and mitotic count. Associations between ITSS grading and categorical variables (necrosis, microvascular proliferation, and molecular markers) were analyzed using the chi-squared test. Additionally, the interobserver variability for ITSS grading was measured using Cohen’s Kappa.

Simple regression analysis was performed to assess the predictive value of radiological parameters (ITSS grade, tumor volume, and rCBV) in determining tumor grade (LGG vs. HGG). Categorical variables (ITSS grade) were encoded as dummy variables. Significant variables from the simple regression analysis were included in a multiple logistic regression model to determine independent predictors of HGG status. Finally, receiver operating characteristic (ROC) curves were generated, and optimal cutoff values were selected based on Youden’s index. Sensitivity, specificity, accuracy, and predictive values for discriminating patients with HGG from those with LGG were calculated by using these cutoff values. ROC curves were compared pairwise using the DeLong test to assess differences in area under the curve (AUC) values.

Statistical analyses were conducted using IBM SPSS Statistics v28.0.1.1 and MedCalc statistical software v23.2.1. *p* < 0.05 was considered to indicate a significant difference.

## 3. Results

### 3.1. Characteristics of the Cohort

Among 113 patients with a pathologically confirmed diagnosis of adult diffuse glioma, 8 were excluded due to incomplete MR imaging, and 6 were excluded because of unavailable molecular results. Therefore, the final study cohort consisted of 99 patients with histologically confirmed adult-type diffuse glioma, with a mean age of 50.3 (standard deviation, 17) years (range, 19–83 years). Most patients were male (n = 52, 52.5%).

Tumors were classified according to WHO CNS5 into three categories: oligodendroglioma, IDH-mutant, and 1p/19q co-deleted (n = 18); astrocytoma, IDH-mutant (n = 38); and glioblastoma, IDH-wildtype (n = 43). All tumors showed a supratentorial location. Patients were stratified using the ITSS grading system (Table 1 and Figure 1).

### 3.2. MRI Interpretation

Semiquantitative ITSS grading correlated positively with rCBV (r_s_ = 0.758; *p* < 0.001) and tumor volume (r_s_ = 0.633; *p* < 0.001). Regarding ITSS classification, the agreement between the two independent observers obtained a Cohen’s Kappa index of 0.822 (0.700–0.944; *p* < 0.001).

### 3.3. Correlation Between Semiquantitative ITSS Grading and Histological and Molecular Findings

**Figure 2:**
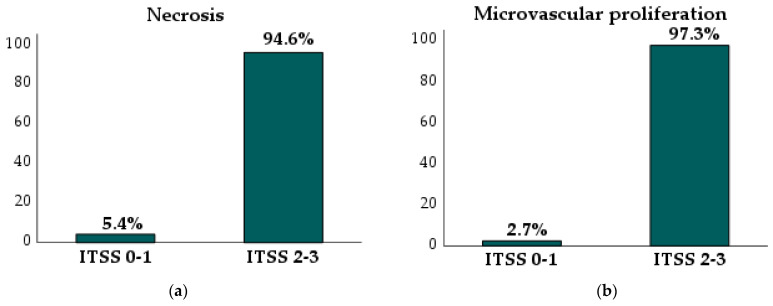
Proportion of necrosis and microvascular proliferation in ITSS grade 0–1 and grade 2–3 gliomas: (**a**) necrosis (*p* < 0.001) and (**b**) microvascular proliferation (*p* < 0.001).

A significant association was found between ITSS grading and tumor type (*p* < 0.001). ITSS grades 0–1 were predominantly observed in oligodendrogliomas (66.7%; *p* < 0.001) and astrocytomas (63.2%; *p* < 0.001), while grades 2–3 were more common in glioblastomas (97.7%; *p* < 0.001). Necrosis and microvascular proliferation showed statistically significant differences across different ITSS grades (*p* < 0.001). Subgroup analysis revealed that necrosis was present in 5.4% of ITSS grade 0–1 gliomas but in 94.6% of ITSS grade 2–3 gliomas (*p* < 0.001) (Figure 2a). Microvascular proliferation occurred in 2.7% of ITSS grade 0–1 gliomas and in 97.3% of ITSS grade 2–3 gliomas (*p* < 0.001) (Figure 2b).

ITSS grading correlated positively with mitotic count (r_s_ = 0.674), the Ki-67 index (r_s_ = 0.697), and the WHO CNS5 tumor grade (r_s_ = 0.835; *p* < 0.001 for all). When segmented by tumor type, the ITSS grading correlation remained positive in both oligodendrogliomas (r_s_ = 0.937; *p* < 0.001) and astrocytomas (r_s_ = 0.624; *p* < 0.001).

**Figure 3:**
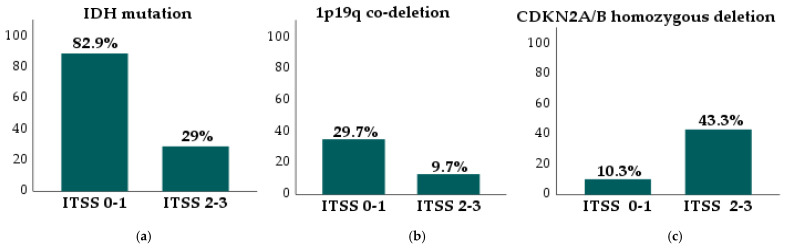
Proportions of IDH mutation, 1p/19q co-deletion, and CDKN2A/B homozygous deletion in ITSS grade 0–1 and grade 2–3 gliomas: (**a**) IDH mutation (*p* < 0.001); (**b**) 1p/19q co-deletion (*p* = 0.01); and (**c**) CDKN2A/B homozygous deletion (*p* = 0.002).

IDH mutations varied across ITSS grades (*p* < 0.001), with mutations being more prevalent in ITSS grades 0–1 (82.9%) compared with grades 2–3 (29%; *p* < 0.001) (Figure 3a). All identified mutations affected the IDH1 protein. The 1p/19q co-deletion status also differed significantly across ITSS grades (*p* = 0.006), with higher frequencies in ITSS grades 0–1 (29.7%) than in grades 2–3 (9.7%; *p* = 0.01) (Figure 3b). CDKN2A/B homozygous deletions showed significant variation across ITSS grades (*p* = 0.015), being more common in grades 2–3 (43.3%) than in grades 0–1 (10.3%; *p* = 0.002) (Figure 3c). This difference remained significant when heterozygous deletions were included in the analysis, with alterations in CDKN2A/B being more prevalent in ITSS grades 2–3 (46.7%) compared with grades 0–1 (13.8%; *p* = 0.021). No significant differences were observed in p53 status across different ITSS grades (*p* = 0.09).

### 3.4. Prediction of Tumor Histological Grade

Univariate regression models demonstrated that ITSS grade, rCBV, and tumor volume (mL) were each significantly associated with glioma grade differentiation (LGG vs. HGG; *p* < 0.001 for all). ROC curve analysis showed an AUC of 0.83 (0.73–0.93; *p* < 0.001) for ITSS, an AUC of 0.85 (0.78–0.93; *p* < 0.001) for rCBV, and an AUC of 0.78 (0.68–0.88; *p* < 0.001) for tumor volume. There were no significant differences in the AUC values between the univariate models: ITSS vs. rCBV (*p* = 0.73), ITSS vs. tumor volume (*p* = 0.479), and rCBV vs. tumor volume (*p* = 0.13).

A multivariable model including ITSS grade, rCBV, and tumor volume did not achieve statistical significance. Consequently, two separate multivariable models were constructed to evaluate independent predictive performance: one incorporating ITSS and rCBV and another combining ITSS and tumor volume. The ITSS + rCBV model yielded an AUC of 0.94 (0.89–0.99; *p* < 0.001) (Table 2), and the ITSS + volume model achieved an AUC of 0.96 (0.92–0.99; *p* < 0.001) (Table 3).

**Figure 4:**
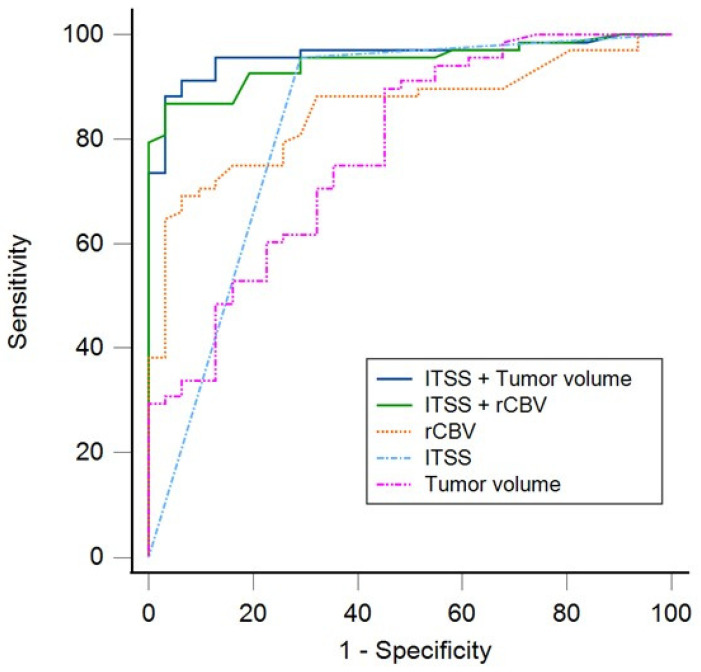
Graphs of the ROC curves for discriminating patients with HGG from those with LGG.

DeLong tests showed a statistically significant increase in the AUC when comparing ITSS + rCBV versus ITSS alone (Δ AUC = 0.12 (0.04–0.19); *p* = 0.004), rCBV alone (Δ AUC = 0.09 (0.03–0.16); *p* = 0.003), and tumor volume alone (Δ AUC = 0.17 (0.06–0.28); *p* = 0.002). The ITSS + tumor volume model also demonstrated a significantly higher AUC compared with ITSS alone (Δ AUC = 0.13 (0.05–0.20); *p* < 0.001), tumor volume alone (Δ AUC = 0.18 (0.08–0.28); *p* < 0.001), and rCBV alone (Δ AUC = 0.11 (0.03–0.18); *p* = 0.007). There were no statistically significant differences in the AUC values between the two multivariable models (*p* = 0.415) (Figure 4).

The optimal cutoff values determined with the Youden Index were 0.64 for the ITSS + rCBV model and 0.68 for the ITSS + volume model. The sensitivity, specificity, accuracy, and predictive values for discriminating patients with HGG from those with LGG are presented in Table 4.

## 4. Discussion

This study underscores the potential of semiquantitative ITSS assessment as a valuable imaging tool for the characterization of adult diffuse gliomas. Our findings reveal a strong correlation between ITSS grading and established diagnostic and prognostic molecular markers in alignment with the WHO CNS5 classification. In addition, our results support the potential role of ITSS in the non-invasive prediction of tumor grade, reinforcing their utility as a relevant imaging biomarker in the management of adult diffuse gliomas.

Regarding imaging analysis, semiquantitative ITSS assessment positively correlated with rCBV and tumor volume, which are well-established markers of higher tumor grade and poorer prognosis [4,5,14,19]. Notably, ITSS grading demonstrated low interobserver variability, supporting its reproducibility and potential for routine clinical application.

Pathological analysis further supports the role of ITSS grading in glioma characterization. ITSS grades 2–3 were found to be more common in IDH-wildtype glioblastomas, while ITSS grades 0–1 were more prevalent in oligodendrogliomas and astrocytomas. In addition, ITSS grades 2–3 gliomas were associated with increased necrosis and microvascular proliferation, reinforcing the value of ITSS as a non-invasive imaging biomarker of tumor biology [2,6,20]. Moreover, semiquantitative ITSS assessment positively correlated with mitotic count and the Ki-67 index, which are hallmark features of aggressive gliomas [6]. Semiquantitative ITSS grading also showed a positive correlation with tumor grade [21]. This correlation persisted within individual tumor types, consistent with the WHO CNS5 classification [2,3]. On the other hand, there were no significant differences in p53 mutation status among the ITSS grades. However, a recent meta-analysis including 23 papers and 2555 patients concluded that the presence of p53 mutations does not provide useful information in the prognosis of glioblastoma, though this represented a significant part of our sample [22].

The WHO CNS5 classification underscores the centrality of molecular markers in glioma diagnosis and prognosis, including IDH mutation, 1p/19q co-deletion, and CDKN2A/B homozygous deletion [2]. In contrast to earlier studies that focused primarily on glioma subtype histological grading or isolated biomarkers, our study integrates semiquantitative ITSS assessment within the diagnostic and molecular framework defined by the WHO CNS5 classification. By correlating ITSS with a comprehensive panel of molecular alterations (including IDH mutation, 1p/19q co-deletion, and CDKN2A/B homozygous deletion), our findings demonstrate the relevance of semiquantitative ITSS grading in the current era of molecularly driven glioma characterization.

Gliomas with ITSS grades 2–3 showed a higher proportion of CDKN2A/B homozygous deletion, which is a well-established predictor of poor prognosis in these tumors [23,24,25,26]. Recent studies indicate that the presence of these molecular alterations is associated with shorter progression-free survival and overall survival in diffuse gliomas [23,25]. Accordingly, in astrocytomas, CDKN2A/B homozygous deletion is now recognized as a molecular criterion for grade 4 designation, even in the absence of histological markers such as necrosis or microvascular proliferation [3]. However, the impact of heterozygous CDKN2A/B deletions remains less definitive, as recent studies suggest that they may not independently affect overall survival in this patient population [23]. To our knowledge, this is the first study to investigate the relationship between ITSS grading and CDKN2A/B deletions, reinforcing the association between high ITSS grades and more aggressive molecular profiles.

On the other hand, IDH1 mutations, a key molecular feature of astrocytomas and oligodendrogliomas, correlate with better prognosis, longer survival, and increased treatment response, particularly to temozolomide [27]. Consistent with prior research, our findings indicate a strong correlation between ITSS grades 0–1 and the presence of IDH1 mutations, highlighting the potential of ITSS assessment in the molecular stratification of diffuse gliomas [10,15,28]. Similarly, 1p/19q co-deletion, a hallmark of oligodendrogliomas associated with favorable prognosis, was notably absent in ITSS grades 2–3, further supporting the link between elevated ITSS and aggressive tumor phenotypes [28,29,30].

Finally, multivariable regression models and ROC analysis demonstrated that incorporating semiquantitative ITSS grading with tumor volume and rCBV significantly enhances the predictive accuracy of these parameters in distinguishing LGG from HGG. This finding aligns with previous studies demonstrating that the integration of ITSS grading with rCBV enhances the accuracy of astrocytoma grading [4]. Both multivariate predictive models demonstrated a great discriminatory performance, with optimal AUCs, high sensitivity, and notably elevated negative predictive values, highlighting their potential in reliably rejecting the probability of high-grade tumors. Although the model including tumor volume achieved slightly higher accuracy, the difference between the two multivariate models did not reach statistical significance. This suggests that, in resource-limited settings where perfusion imaging is unavailable, tumor volume may serve as a feasible complementary parameter to ITSS for non-invasive glioma grade prediction. However, volumetric markers remain less validated than rCBV analysis and should be interpreted with appropriate consideration until further prospective validation is available.

The use of ITSS as a semiquantitative imaging marker offers practical clinical value, particularly in preoperative glioma assessment. ITSS grading can be implemented without the need for advanced post-processing, making it accessible even in resource-limited settings. In routine practice, semiquantitative ITSS assessment may aid in the non-invasive prediction of tumor grade and support surgical planning, especially when molecular diagnostics or perfusion imaging are not readily available. Taken together, our findings suggest that diffuse gliomas with ITSS grades 2–3 exhibit biological and molecular characteristics consistent with those of high-grade tumors and should be evaluated accordingly. When combined with rCBV and tumor volume, ITSS grading enhances diagnostic accuracy and may serve as a valuable tool in preoperative risk stratification and treatment planning. This semiquantitative, non-invasive marker aligns with the WHO CNS5 framework and may help guide clinical decisions in cases where histological or molecular data are pending or unavailable.

As a retrospective, single-center study, future multicenter studies with broader population samples are warranted to validate our results. The limited sample size prevented the stratification of the multivariate analysis regarding tumor grade across different tumor types. Although the overall cohort size was acceptable, we acknowledge that subgroup analyses, especially those involving less prevalent molecular alterations such as CDKN2A/B deletions (n = 32), may be constrained by limited sample representation and should be further investigated. Finally, in our study, ITSS grading remained semiquantitative instead of quantitative [12,31]. This choice was based on its established clinical utility, interpretability, and reproducibility in the prior literature, prioritizing clinical feasibility [4,8,10,16]. Although interobserver agreement was high, we acknowledge that future studies should explore the use of voxel-wise or AI-driven segmentation methods to validate and potentially refine ITSS-based markers in larger external cohorts.

## 5. Conclusions

Our findings suggest that diffuse gliomas with ITSS grades 2–3 should be regarded as high-grade tumors, especially when supported by additional parameters such as elevated rCBV and increased tumor volume. These gliomas demonstrated a higher prevalence of CDKN2A/B homozygous deletions, an established predictor of poor prognosis in these tumors, and a lower proportion of favorable markers, including IDH mutations and 1p/19q co-deletions. Furthermore, they exhibited a greater prevalence of histopathological features of aggressiveness, including microvascular proliferation and necrosis. Taken together, semiquantitative ITSS grading provides a non-invasive imaging biomarker that reflects both molecular and pathological tumor profiles, aligning with the WHO CNS5 classification and offering valuable clinical insights in glioma diagnosis and management.

## Figures and Tables

**Table 1:** Descriptive analysis of imaging, histopathological, and molecular features across different ITSS grades in adult diffuse gliomas.

Characteristics		ITSS Grade			
		Grade 0 (n = 12)	Grade 1 (n = 25)	Grade 2 (n = 16)	Grade 3 (n = 46)
**Imaging features**					
rCBV ^1^		1.1 (0.8–1.2)	1.6 (1–1.9)	2.4 (1.2–2.5)	5.3 (4.1–6.1)
Volume in mL ^1^		3.1 (1.3–3.2)	15.9 (2–27.3)	19.4 (4.2–20.4)	43.6 (16.7–62.2)
**Tumor type**					
Oligodendroglioma ^2^		1 (8.3%)	11 (44%)	2 (12.5%)	4 (8.7%)
Astrocytoma ^2^		11 (91.7%)	13 (52%)	10 (62.5%)	4 (8.7%)
Glioblastoma ^2^		0 (0%)	1 (4%)	4 (25%)	38 (82.6%)
**Glioma grade**					
Low grade	Grade 1 ^2^	6 (50%)	3 (12%)	0 (0%)	0 (0%)
	Grade 2 ^2^	3 (25%)	19 (76%)	0 (0%)	0 (0%)
High grade	Grade 3 ^2^	3 (25%)	1 (4%)	11 (68.8%)	6 (13%)
	Grade 4 ^2^	0 (0%)	2 (8%)	5 (31.2%)	40 (87%)
**Morphological features**					
Mitosis/10 HPF ^1,3^		0 (0–4)	2 (0–3)	7 (4–12)	14 (6–20)
Microvascular proliferation ^2^		0 (0%)	1 (4%)	3 (18.8%)	38 (82.6%)
Necrosis ^2^		1 (8.3%)	1 (4%)	4 (25%)	42 (91.3%)
**Immunohistochemistry**					
Ki-67 index ^1^		3.75 (1–5)	7.24 (3–8)	27.00 (10–40)	31.17 (20–40)
p53 mutation ^2^		6 (50%)	13 (52%)	10 (62%)	14 (30.4%)
**Molecular analysis**					
IDH mutation ^2^		7 (58.3%)	17 (68%)	8 (50%)	6 (13.3%)
1p/19q co-deletion ^2^		1 (8.3%)	10 (40%)	2 (12.5%)	4 (8.7%)
CDKN2A/B deletion		1 (11.1%)	3 (15%)	6 (40%)	22 (49.9%)
*Homozygous deletion* ^2^		1 (11.1%)	2 (10%)	5 (33.3%)	21 (46.7%)
*Heterozygous deletion* ^2^		0 (0%)	1 (5%)	1 (6.7%)	1 (2.2%)

^1^ Median (IQR), ^2^ n (% among ITSS grade), ^3^ HPF (high-power field).

**Table 2:** Logistic regression model’s prediction of glioma grade based on ITSS and rCBV.

Variable	Coefficient (β)	OR ^1^ (95%CI)	*p* Value
ITSS	−3.388	0.034 (0.007–0.158)	*p* < 0.001
rCBV	0.87	2.387 (1.365–4.172)	*p* = 0.002
Constant	−0.242	0.785	*p* = 0.695

^1^ OR: Odds ratio.

**Table 3:** Logistic regression model’s prediction of glioma grade based on ITSS and tumor volume.

Variable	Coefficient (β)	OR ^1^ (95%CI)	*p* Value
ITSS	−4.605	0.010 (0.001–0.074)	*p* < 0.001
Tumor volume	0.081	1.085 (1.024–1.149)	*p* = 0.006
Constant	0.677	1.968	*p* = 0.142

^1^ OR: Odds ratio.

**Table 4:** Cutoff values for the probability of high-grade glioma in predictive models.

Model	Cutoff	SE	Sp	ACC	PPV	NPV	AUC (95%CI)
ITSS + rCBV	0.64	95.2%	71.4%	80.8%	71.4%	95.2%	0.94 (0.89–0.99)
ITSS + volume	0.68	95.6%	74.1%	87.8%	71%	95.8%	0.96 (0.92–0.99)

SE: sensitivity; Sp: specificity; ACC: accuracy; PPV: positive predictive value; NPV: negative predictive value; AUC: area under the curve.

## Data Availability

The dataset supporting the findings of this study is available in the Figshare repository at https://doi.org/10.6084/m9.figshare.29038580 (accessed on 2 June 2025). A minimal dataset was also provided during the submission process for editorial review.

## Abbreviations

The following abbreviations are used in this manuscript.

AUC	Area under the curve
CDKN2A/B	Cyclin-dependent kinase inhibitors 2A and 2B
DSC	Dynamic susceptibility contrast
HGG	High-grade glioma
IDH	Isocitrate dehydrogenase
ITSS	Intratumoral susceptibility signals
LGG	Low-grade glioma
rCVB	Relative cerebral blood volume
ROC	Receiver operating characteristic
SWAN	Susceptibility-weighted angiography
SWI	Susceptibility-weighted imaging
WHO CNS5	Fifth edition of the World Health Organization Classification of Tumors of the Central Nervous System
